# Syndrome de Williams-Beuren: étude rétrospective d’une série de 11 cas du Centre Hospitalier Universitaire Mohammed VI de Marrakech

**DOI:** 10.11604/pamj.2023.46.94.29604

**Published:** 2023-12-01

**Authors:** Fatima Zahrae Bouzid, Hanane Ait Hammou, Hassan Akallakh, Kenza Dafir, Mariam Tajir, Nisrine Aboussair

**Affiliations:** 1 Service de Génétique, Centre de Recherche Clinique, Centre Hospitalier Universitaire Mohammed VI, Marrakech, Maroc,; 2 Faculté de Médecine et de Pharmacie de Marrakech, Université Cadi Ayyad, Marrakech, Maroc,; 3 Service de Génétique Médicale, Centre Hospitalier Universitaire Mohammed VI, Oujda, Maroc

**Keywords:** Williams-Beuren, 7q11.23, FISH, elastine, Williams-Beuren syndrome, 7q11.23, FISH, elastin gene

## Abstract

Le syndrome de Williams-Beuren est une maladie génétique rare (1/20 000). Il s'agit d'une microdélétion en 7q11.23 sporadique dans la majorité des cas regroupant 28 gènes dont celui de l'élastine (ELN). Facilement identifiable dans l'enfance, cette anomalie du développement associe une dysmorphie faciale évocatrice, une malformation cardiaque, un retard psychomoteur, un profil comportemental et cognitif spécifique. Nous rapportons dans ce travail une étude rétrospective de 11 cas du syndrome de Williams-Beuren colligée au Service de Génétique du CHU Mohammed VI de Marrakech, l'âge moyen des patients est de 6,05 ans (SD= 6,56; interquartile range= 5), avec une prédominance du sexe féminin 64% (7/11). Le retard mental était quasi constant chez tous nos patients, le diagnostic est confirmé chez 100% (11) des patients par la technique d'hybridation in situ en fluorescence (FISH).

## Abstract (en)

Williams-Beuren syndrome is a rare genetic disease (1/20 000) characterized by a microdeletion at 7q11.23 encompassing about 28 genes, including the elastin gene, ELN. It is a sporadic disease in the majority of cases. Easily identifiable in childhood, this developmental disorder associates suggestive face dysmorphism, cardiac defect, psychomotor retardation and specific behavioural and cognitive profile. We conducted a retrospective study of 11 patients with Williams-Beuren syndrome whose data were collected in the Genetics Department of the Mohammed VI University Hospital of Marrakech. The average age of patients was 6.05 years (SD=6.56; interquartile range=5), with a female predominance (64%; 7/11 patients). Almost all patients were mentally retarded and the diagnosis was confirmed in 100% (11) of patients using fluorescence in situ hybridisation (FISH).

## Introduction

Le syndrome de Williams-Beuren (SWB) est une maladie génétique rare et sporadique dans la majorité des cas dont l'incidence des formes typiques à la naissance est de 1/20 000 [1,2]. Deux cardiologues pédiatres, Williams et Beuren, ont décrit indépendamment en 1961 et 1962 un syndrome associant de manière assez homogène un retard psychomoteur avec des difficultés d'apprentissage, une dysmorphie apparentée à un faciès d'elfe, un comportement amical caractéristique, et une cardiopathie de type [3,4]. Il s'agit d'un trouble du développement qui associe une dysmorphie faciale typique avec des malformations cardiovasculaires, un profil neuropsychologique et comportemental spécifique caractérisé par une hypersociabilité, des troubles du langage, des déficits visiospatiaux et un retard cognitif modéré à sévère. En 1993, Ewart *et al*., ont identifié l'anomalie génétique impliquée dans le syndrome de Williams-Beuren, il s'agit d'une microdélétion submicroscopique située dans la région q11.23 de l'un des deux chromosomes 7 [5]. Cette anomalie est présente dans 95 % des cas de SWB [6]. Quant aux autres patients chez qui cette microdélétion n'a pas été mise en évidence, il s'agit probablement d'une microdélétion de taille inférieure au seuil de détection ou bien d'autres anomalies chromosomiques qui entraînent un phénotype semblable à celui du SWB.

Le SWB est un syndrome des gènes contigus car les manifestations cliniques résultent de l'implication de plusieurs gènes, chacun est responsable d'une partie du phénotype. La région délétée mesure environ 1,5 Mb d'ADN. Elle comprend le gène codant pour l'élastine [7]. Le gène de l'élastine (ELN) est localisé sur la région proximale du bras long du chromosome 7 en 7q11.23, aux environs du centre de la délétion [8]. Il mesure 50 kb et est composé de 34 exons de petite taille (27 à 165 pb). C'est le seul gène qui est lié sans équivoque au phénotype du SWB, provoquant la SASV et d'autres sténoses vasculaires [9]. L'ELN est le constituant principal de la matrice extracellulaire de la paroi vasculaire, elle intervient dans le développement de la paroi artérielle en jouant un rôle dans la prolifération des cellules musculaires lisses vasculaires [1]. En plus de l'élastine, la délétion inclut au moins 28 autres gènes [10] tels que LIMK1, GTF21 et STX1A [11]. Notre travail est la première étude sur le syndrome de Williams-Beuren dans la région du Marrakech et nous faisons le point sur ce syndrome génétique. L'objectif de notre étude est de montrer l'intérêt du généticien dans le diagnostic des syndromes rares et le conseil génétique adéquat des patients et leurs familles.

## Méthodes

**Type et paramètres de l'étude:** il s'agit d'une étude rétrospective menée au CHU Mohammed VI, un établissement universitaire public localisé à Marrakech. Tous les patients avaient bénéficié d'un examen dysmorphologique minutieux associé à une étude généalogique.

**Participants:** onze cas du syndrome de Williams-Beuren, colligés en consultation de génétique du CHU Mohammed VI de Marrakech par des pédiatres et cardiologues pour une dysmorphie faciale associée à un retard des acquisitions psychomotrices pour diagnostic et conseil génétique adéquat.

**Collecte de données:** les données de l'étude généalogique, de l'examen clinique (dysmorphologique et comportemental) et du bilan malformatif chez nos patients ont été collectées (d'avril 2008 à janvier 2022) et nous ont permis d'évoquer le diagnostic du SWB (Tableau 1).

**Tableau 1:** récapitulatif des données généalogiques et des manifestations cliniques et paracliniques observées chez nos patients

	P1	P2	P3	P4	P5	P6	P7	P8	P9	P10	P11
Sexe	F	M	F	M	M	F	F	F	M	F	F
Age	8mois	6ans	2ans	2ans	1an	3 ans	6ans	9ans	4 ans	8 ans	25
PN	2kg 900	2kg 500	3kg500	2kg 800	1kg 300	2kg 900	3kg 100	3kg	-		3kg
Age maternel	26	34	32	32	37	23	35	37	-	39	49
Dysmorphie	+	+	+	+	+	+	+	+	+	+	+
Atteinte cardiaque	CIA	-	-	CIA haute	CIA	CIA	IA	CIA+CIV	CIA	Sténose aortique supra valvulaire	Sténose aortique supravalvulaire
Hyper sociabilité	+	+	+	+	+	+	+	+	+	+	+
Retard mental	léger	sévère	léger	sévère	léger	léger	modéré	sévère	modérée	sévère	sévère
Atteinte dentaire	diastasis	Mauvais état bucco-dentaire	-	diastasis	-	-	caries	Caries	Mauvais état bucco-dentaire	-	Caries
Atteinte ophtalmologique	-		-		-	En cours	Isotropie oeil gauche	-	En cours	-	-
Calcémie			hypercalcémie	hypercalcémie	En cours	-	hypercalcémie	-	-	-	-

**Figure 1:**
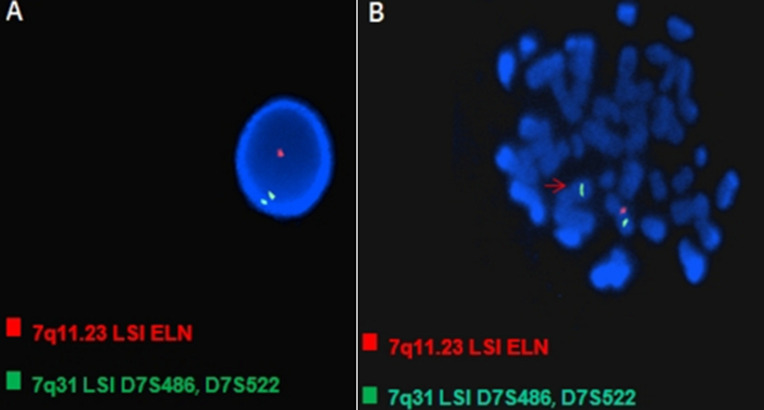
FISH interphasique mettant en évidence la microdélétion hétérozygote en 7q11.23; A) deux spots verts témoins des deux chromosomes 7 et un seul spot rouge témoin du gène de l'élastine sur noyaux colorés par le DAPI; B) la flèche montre l'absence du spot rouge sur un chromosome 7 métaphasique confirmant ainsi la délétion hétérozygote du gène de l'élastine

**Analyse de laboratoire:** la technique d'hybridation in situ en fluorescence après culture cellulaire réalisée à partir de lymphocytes circulants en métaphase (Figure 1) a été réalisée chez tous les patients et a objectivé la présence d'une microdélétion hétérozygote en 7q11.23 responsable du SWB et dont la formule chromosomique est la suivante : ishdel (7)(q11.23q11.23)(ELN/D7S613). Le principe général de la technique est fondé sur la propriété de réassociation des acides nucléiques, une sonde dénaturée (ADN simple brin marqué) en solution peut s'hybrider spécifiquement avec sa séquence cible (préparation chromosomique dénaturée) grâce à la complémentarité des bases, La sonde s'apparie par des liaisons hydrogènes établies.

**Analyses statistiques:** l'analyse statistique a été réalisée à l'aide du Microsoft Office Excel.

**Les considérations éthiques:** pour chaque patient, l'objet et la démarche de la recherche ont été clairement expliqués. Leur consentement était recueilli à l'aide de la notice d'information et de la fiche du consentement éclairé.

## Résultats

### Caractéristiques cliniques et génétiques

La grossesse et l'accouchement étaient sans anomalies notamment pas de notion de prise d'un traitement tératogène ou de souffrance néonatale chez tous nos patients. Le poids de naissance était inférieur à 3kg dans 37% des cas, entre 2kg et 2kg500 dans 50% des cas et moins de 2kg dans 13% des cas. Par ailleurs, l'âge moyen de la découverte du syndrome est de 6 ans dans notre série. En outre le retard des acquisitions psychomotrices est noté chez 100 % de nos patients avec un âge moyen de la marche de 2 ans et demi alors que celui de la parole est de 3 ans après rééducation orthophonique. Dans notre série de 11 patients il y'avait une prédominance du sexe féminin avec un pourcentage de 64%; 81% Des patients ont une maladie cardio vasculaire; Le retard mental était quasi constant chez tous nos patients. La technique d'hybridation in situ en fluorescence (FISH) a confirmé le diagnostic chez la totalité de nos patients, par la mise en évidence d'une microdélétion hétérozygote en 7q11.23 responsables du SWB. La formule chromosomique est la suivante: ishdel (7) (q11.23q11.23)(ELN/D7S613).

**Prise en charge:** nos patients ont bénéficié d'un suivi multidisciplinaire faisant intervenir des pédiatres, des cardiologues, des chirurgiens, des psychomotriciens, des orthophonistes, des psychologues, des dentistes, des ophtalmologues, des endocrinologues et des généticiens. De même une prise en charge à vie s'est avérée nécessaire.

## Discussion

L'objectif de notre étude est de montrer le rôle de la génétique dans e diagnostic des maladies rares comme le syndrome de Williams-Beuren. On a confirmé le diagnostic chez les 11 patients de notre cohorte. Les anomalies cardiovasculaires sont présentes chez 75% des patients. Les anomalies caractéristiques sont la sténose aortique supravalvulaire (SASV) et les sténoses des branches de l'artère pulmonaire. Une atteinte des valves aortiques ou mitrales défectueuses, une tétralogie de Fallot peuvent être diagnostiquées dès le jeune âge [12] et pouvant être responsables d'accidents ischémiques cardiaques ou cérébraux [13]. En plus, 9 patients parmi les 11 de notre série présentent une atteinte cardiaque (Tableau 1). Sur le plan systémique, d'autres atteintes sont fréquentes: une hypercalcémie néonatale, des troubles digestifs de la petite enfance et des anomalies ophtalmologiques (presque 40% des malades présentent un strabisme ou des troubles de la réfraction) [14]. Un retard de croissance postnatal avec une taille en dessous du 10ème percentile et une microcéphalie modérée. Une surveillance de la fonction thyroïdienne est importante car l'hypothyroïdie n'est pas rare [15]. De même, on note parfois une intolérance glucidique, des manifestations articulaires et des anomalies buccodentaires [16,17]. Parmi les anomalies dentaires on note des dents de lait petites, irrégulières et espacées, des caries et une hypoplasie de l'émail [18].

Une échographie rénale peut dépister des malformations urétéro-rénales qui sont fréquentes chez les sujets avec SWB. La recherche systématique d'hypercalcémie et une surveillance de la fonction rénale sont indiquées car une néphrocalcinose et une insuffisance rénale peuvent apparaitre avec l'âge, dans notre étude, 27% de patients présentaient une hypercalcémie. De même une sténose des artères rénales à l'origine d'une hypertension réno-vasculaire, peut être présente dès la naissance. Il est à noter que 27% de nos patients présentaient une hypercalcémie.

Parmi les anomalies osteo-articulaires observées chez les patients présentant un SWB: une attitude caractéristique avec des épaules tombantes, une hyperlordose lombaire et un flexum des hanches et des genoux. Chez 17% des cas une scoliose peut survenir, et chez 15% des cas une ankylose des grosses articulations est observée. Une synostose radio-cubitale à l'origine d'une limitation de la supination du coude est observée dans 10% des cas, causant des difficultés pour dessiner, écrire et manipuler les objets. En outre, une luxation récidivante des rotules chez 5 % des cas [12] ainsi qu'une déformation du thorax en pectus excavatum. Dans notre étude, aucun cas d'atteinte squelettique n'a été observé. Sur le plan neurocognitif et psychologique, les individus avec SWB présentent un profil cognitif particulier, des capacités exceptionnelles dans certains domaines et un retard important dans d'autres. Ils présentent un comportement hypersociable. Néanmoins, de nombreuses études notent des troubles anxieux ainsi qu'une hypersensibilité au bruit dès la petite enfance conduisant à une attirance marquée pour la musique, une dissociation entre un déficit cognitif et une fonction linguistique relativement bien préservée, et des repères visiospatiaux déficitaires [12].

Devant toute suspicion clinique du SWB, la mise en évidence de l'anomalie en cause doit être recherchée. Cette anomalie cytogénétique n'est pas visible sur un caryotype standard, mais mise en évidence par la technique d'hybridation in situ en fluorescence (FISH) utilisant une sonde spécifique de la région 7q11.23. La microdélétion impliquée survient le plus souvent de novo, d'où le caractère sporadique de cette affection [7]. Dans de rares cas, elle est héritée d'un parent suivant un mode autosomique dominant. Tous les cas répertoriés dans notre étude sont des cas sporadiques, puisque les parents sont phénotypiquement normaux et il n'y pas d'indication d'explorer les parents par FISH (il n'y a jamais eu de cas rapporté de microdélétion en 7q11.23 avec un phénotype normal) [8]. Le risque de récurrence d'un Syndrome de Williams chez les parents de nos patients est très faible mais non nul, étant donné le risque de mosaïcisme germinal. Toutes les familles de nos patients ont bénéficié d'un conseil génétique adéquat. Les couples ayant donné naissance à un enfant atteint peuvent bénéficier, s'ils le souhaitent, d'un diagnostic prénatal (DPN) lors d'une grossesse ultérieure. L'objectif du DPN est de déterminer au cours de la grossesse, si l'enfant à naître est atteint ou non du syndrome. Il consiste à rechercher l'anomalie chromosomique, sur un prélèvement fait au niveau du futur placenta (choriocentèse) ou du liquide amniotique (amniocentèse).

Le pronostic de la pathologie dépend de l'atteinte cardiaque et l'espérance de vie est inconnue. Le suivi des personnes atteintes du SWB varie selon l'âge du diagnostic et l'importance de chacune des manifestations présentes. Dans la plupart des cas, cette prise en charge sera complexe et pluridisciplinaire associant pédiatres, cardiopédiatres, orthodontistes, orthopédistes, ophtalmologues, psychomotriciens, orthophonistes, endocrinologues, psychologues et généticiens. Des règles de suivi médical ont été données par l'Académie américaine de pédiatrie [8]. Tous nos patients ont bénéficié d'une prise en charge pluridisciplinaire.

## Conclusion

Le syndrome de Williams-Beuren est une maladie génétique rare due à une microdélétion hétérozygote en 7q11.23 qui emporte le gène de l'élastine. Le diagnostic est évoqué devant la dysmorphie faciale caractéristique. De même, ce diagnostic est facile à confirmer par une technique d'hybridation in situ en fluorescence (FISH) recherchant la microdélétion en 7q11.23. L'atteinte cardiaque est la plus grave et nécessite un suivi spécialisé. Il existe des formes partielles auxquelles il faut penser devant tout phénotype comportemental spécifique. Dans notre étude, le diagnostic de SWB est confirmé chez 100% (11) des patients par la technique d'hybridation in situ en fluorescence (FISH).

## Remerciements

Nous souhaitons remercier le personnel du Laboratoire de Génétique Médicale de Marrakech, ainsi que tous les médecins qui nous ont référé les patients, pour leur coopération et leur soutien au cours de cette étude.

## References

[1] Ewart AK, Jin W, Atkinson D, Morris CA, Keating MT (1994). Supravalvular aortic stenosis associated with a deletion disrupting the elastin gene. J Clin Invest.

[2] Stromme P, Bjornstad PG, Ramstad K (2002). Prevalence estimation of Williams syndrome. J Child Neurol.

[3] Williams JC, Barratt-Boyes BG, Lowe JB (1961). Supravalvular aortic stenosis. Circulation.

[4] Beuren AJ, Apitz J, Harmjanz D (1962). Supravalvular aortic stenosis in association with mental retardation and a certain facial appearance. Circulation.

[5] Ewart AK, Morris CA, Atkinson D, Jin W, Sternes K, Spallone P (1993). Hemizygosity at the elastin locus in a developmental disorder, Williams syndrome. Nature Genet.

[6] Nickerson E, Greenberg F, Keating MT, McCaskill C, Shaffer LG (1995). Deletions of the elastin gene at 7q11.23 occur in approximately 90% of patients with Williams syndrome. Am J Hum Genet.

[7] Meng X, Lu X, Morris CA, Keating MT (1998). A novel human gene FKBP6 is deleted in Williams syndrome. Genomics.

[8] Lacroix A, Pezet M, Capel A, Bonnet D, Hennequin M, Jacob MP (2009). Gilbert-Dussardier. Le syndrome de WilliamsBeuren?: une approche pluridisciplinaire.

[9] Li DY, Toland AE, Boak BB, Atkinson DL, Ensing GJ, Morris CA (1997). Elastin point mutations cause an obstructive vascular disease, supravalvular aortic stenosis. Hum Mol Genet.

[10] Lowery MC, Morris CA, Ewart A, Brothman LJ, Zhu XL, Leonard CO (1995). Strong correlation of elastin deletions, detected by FISH, with Williams syndrome: evaluation of 235 patients. Am J Hum Genet.

[11] Howald C, Merla G, Digilio MC (2006). Two high throughput technologies to detect segmental aneuploidies identify new Williams-Beuren syndrome patients with atypical deletions. J Med Genet.

[12] Eronen M, Peippo M, Hiippala A, Raatikka M, Arvio M, Johansson R (2002). Cardiovascular manifestations in 75 patients with Williams syndrome. J Med Genet.

[13] Vaideeswar P, Shankar V, Deshpande JR, Sivaraman A, Jain N (2001). Pathology of the diffuse variant of supravalvar aortic stenosis. Cardiovasc Pathol.

[14] Martin ND, Smith WR, Cole TJ, Preece MA (2007). New height, weight and head circumference charts for British children with Williams syndrome. Arch Dis Child.

[15] Cambiaso P, Orazi C, Digilio MC, Loche S, Capolino R, Tozzi A (2007). Thyroid morphology and subclinical hypothyroidism in children and adolescents with Williams syndrome. J Pediatr.

[16] Metcalfe K (1999). Williams syndrome: an update on clinical and molecular aspects. Arch Dis Child.

[17] Cherniske EM, Carpenter TO, Klaiman C, Young E, Bregman J, Insogna K (2004). Multisystem study of 20 older adults with Williams syndrome. Am J Med Genet A.

[18] Axelsson S, Bjørnland T, Kjaer I, Heiberg A, Storhaug K (2003). Dental characteristics in Williams syndrome: a clinical and radiographic evaluation. Acta Odontol Scand.

